# Clarifying the Biomechanical Concept of Coordination Through Comparison With Coordination in Motor Control

**DOI:** 10.3389/fspor.2021.753062

**Published:** 2021-10-14

**Authors:** Arata Kimura, Toshiharu Yokozawa, Hiroki Ozaki

**Affiliations:** Department of Sports Research, Japan Institute of Sports Sciences, Tokyo, Japan

**Keywords:** biomechanics, conceptual analysis, coordination, motor control, performance enhancement

## Abstract

Coordination is a multidisciplinary concept in human movement science, particularly in the field of biomechanics and motor control. However, the term is not used synonymously by researchers and has substantially different meanings depending on the studies. Therefore, it is necessary to clarify the meaning of coordination to avoid confusion. The meaning of coordination in motor control from computational and ecological perspectives has been clarified, and the meanings differed between them. However, in biomechanics, each study has defined the meaning of the term and the meanings are diverse, and no study has attempted to bring together the diversity of the meanings of the term. Therefore, the purpose of this study is to provide a summary of the different meanings of coordination across the theoretical landscape and clarify the meaning of coordination in biomechanics. We showed that in biomechanics, coordination generally means the relation between elements that act toward the achievement of a motor task, which we call biomechanical coordination. We also showed that the term coordination used in computational and ecological perspectives has two different meanings, respectively. Each one had some similarities with biomechanical coordination. The findings of this study lead to an accurate understanding of the concept of coordination, which would help researchers formulate their empirical arguments for coordination in a more transparent manner. It would allow for accurate interpretation of data and theory development. By comprehensively providing multiple perspectives on coordination, this study intends to promote coordination studies in biomechanics.

## Introduction

Coordination is one of the central concepts in human movement science, especially in the field of biomechanics and motor control. Then, is there a common idea of coordination in these fields? Even though we believe there are more similarities than most people appreciate, there may be differences as well. Therefore, this study will focus on the denotation and connotation of coordination in these fields.

Biomechanics is a field that aims to identify the function of elements (e.g., muscles, joint movements) involved in achieving motor tasks. This identification is expected to provide information on improving movement and making it safer. However, because the musculoskeletal system is highly interconnected and integrated, it is almost impossible to identify the function of each element in isolation during a whole-body movement. Therefore, coordination between elements should be taken into account (Winter, 2009). In recent years, there has been a growing interest in coordination, as seen by the addition of a chapter regarding coordination in a well-known textbook on biomechanics, which was not included in the first edition (Winter, 2009; Robertson et al., 2013).

In terms of coordination, one central question in motor control is how a large number of degrees of freedom in the human body become organized (Bernstein, 1967). The human body has ~300 joints and 800 muscles, which combined bring about changes in the overall movement of the body. For the production of a successful movement, it is believed that the different degrees of freedom at each spatiotemporal scale should be coordinated. This issue has been examined from multiple perspectives in motor control, the most typical of which are computational and ecological perspectives (Bruton and O'dwyer, 2018; Profeta and Turvey, 2018). The computational perspective is a branch of motor control that views the brain as a computational machine and attempts to understand how the central nervous system (CNS) processes information to move the body. The researchers in a computational perspective conclude that a large number of degrees of freedom are coordinated by the control of the CNS (d'Avella and Bizzi, 1998; Saltiel et al., 2001; d'Avella et al., 2003). The ecological perspective is a branch of motor control that attempts to understand human movement through the interaction between the human body and its surrounding environment, situation, and context, without giving the CNS a privileged status. The researchers in the ecological perspective conclude that coordination takes place without intervention by an external directing control, such as the CNS (Kugler et al., 1980; Turvey, 1990, 2007); thus, the coordination between a large number of degrees of freedom is not pre-designed but emerges spontaneously.

It turns out that the term coordination has been examined in a variety of contexts. Such a term tends to be often not used synonymously and has different meanings because the meaning of a concept is context-dependent. Using ambiguously defined terms may lead to communicative conflicts and misunderstandings. Therefore, clarification of the meaning of terms is necessary to avoid confusion. Previous studies clarified the meaning of coordination in motor control in terms of computational and ecological perspectives and indicated that the meanings differed between them (Bruton and O'dwyer, 2018; Profeta and Turvey, 2018). However, in biomechanics, each study has defined the meaning of the term and the meanings are diverse, but no study has attempted to bring together the diversity of meanings of the term. Therefore, the meaning of coordination in biomechanics remains ambiguous and it may prevent the progress of coordination studies in biomechanics.

The purpose of this study is to provide a summary of the different meanings of coordination across the theoretical landscape and clarify the meaning of coordination in biomechanics. Through this, we intend to establish a biomechanical perspective on coordination. In the present study, we emphasize coordination in terms of biomechanics, but we believe that this study has implications for various other related fields. Although coordination has been studied in various fields, researchers in different fields are not yet aware of each other's work. Therefore, this study will focus on summarizing the basic findings commonly shared in each field rather than summarizing the latest findings. We thought that we should first summarize the basic findings of each field and then combine these findings in order to help researchers in the various fields. We hope to enhance our understanding of each other's work and suggest useful directions for future progress.

The remaining of this paper consists of five sections. Section Meaning of coordination in biomechanics deals with coordination in biomechanics. Section Meaning of coordination in motor control relates to the coordination used in motor control, particularly from computational and ecological perspectives. Section Comparison across fields focuses on the similarities and differences in the meaning of coordination between biomechanical and computational perspectives, and biomechanical and ecological perspectives. Section Some topics to be discussed presents some topics for further study regarding coordination in biomechanics. Finally, Section Conclusion provides the significance of this study.

## Meaning of Coordination in Biomechanics

The term coordination is not used synonymously and has substantially different meanings in biomechanics. There are at least two different meanings of coordination. One views coordination as the relation between elements to achieve a common task goal. The other views coordination as an interrelation of multiple elements and does not explicitly include acting toward a common goal. In this section, we discuss the different meanings of coordination in more detail.

### Functional Relationships for the Achievement of a Motor Task

The most prominent definition of coordination relates to the functional relationships between elements for the achievement of a motor task. Zatsiorsky and Prilutsky (2012) defined muscle coordination as the distribution of muscle activation or force among individual muscles to produce a given motor task. Winter (2009) used the term synergy rather than coordination and defined synergy as elements collaborating toward a common goal. Zajac et al. (2002) also defined the term synergy as co-acting elements to achieve a task goal that is unobtainable by one element alone. These definitions commonly imply that coordination or synergy means the relation between elements to achieve a motor task, called biomechanical coordination in this study.

**Figure 1:**
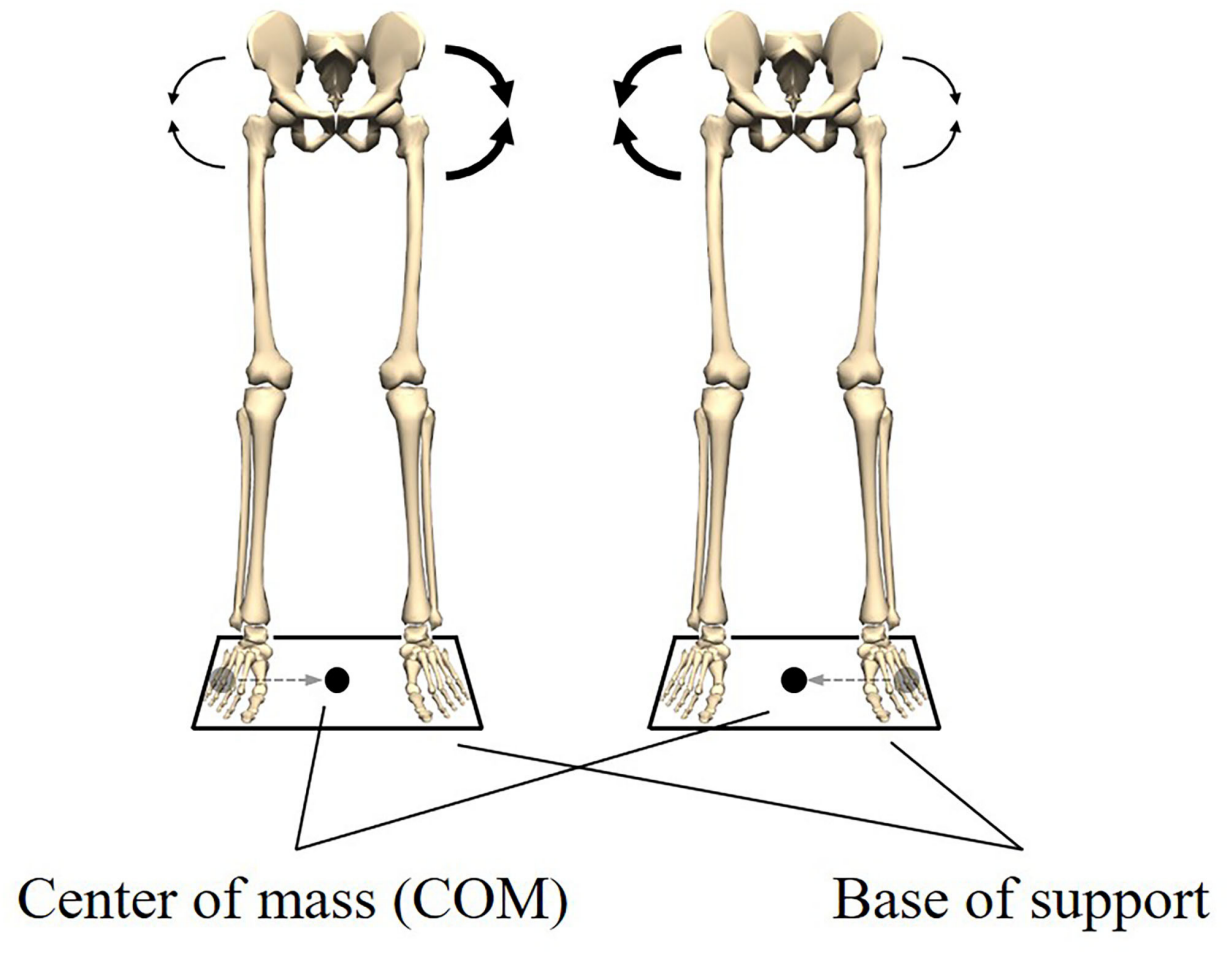
Postural stability in the left-right direction during quiet standing. Keeping the center of mass (COM) within the base of support is a functional requirement to maintain quiet standing. The functional requirement is met by the coordination of the left and right hip abductors/adductors moments. When the COM is located at the left end of the base of support, the right abductor moment increases and the left abductor moment decreases. Also, when the COM is located at the right end of the base of support, the left abductor moment increases and the right abductor moment decreases. This relationship between the moments allows the COM to be kept within the base of support.

To form an image, the meaning of biomechanical coordination is illustrated using quiet standing with both legs. In quiet standing, the center of mass (COM) should be kept within the base of support to maintain posture (Shumway-Cook and Horak, 1986; Kuo, 1995; Hof et al., 2005). In other words, keeping the COM within the base of support is a functional requirement to maintain quiet standing. Here, for the sake of simplicity, we focus only on the position of the COM in the left-right direction. Using two force platforms and performing inverse dynamics, it was found that the position of the COM was controlled by the left and right hip abductor/adductor moments (Winter et al., 1996). Furthermore, the left and right hip abductor/adductor moments were exactly equal in magnitude and 180° out of phase (Winter et al., 1996). These results indicated that an increased abductor moment on one side was accompanied by decreased abductor moments on the contralateral side (Figure 1). The relationship between these moments led to a stable left-right balance and quiet standing was maintained. It can, thus, be concluded that these moments were coordinated.

As the definition suggests, biomechanical coordination has a fairly inclusive meaning. Therefore, several terms are similar to biomechanical coordination: “cooperation,” “collaboration,” and “compensation.” These terms, of course, have broader meanings. For instance, cooperation usually implies association for common benefit; collaboration often connotes working together to achieve something; compensation usually implies the correction of loss. Although these words have their own connotations, an important part of each involves the relation between elements to achieve a common goal. It is sometimes useful to consider all these terms as different forms of biomechanical coordination.

To identify biomechanical coordination, at least the following two factors should be met, as also noted by Latash and Zatsiorsky (2015). First, the elements must be related rather than independent, i.e., the elements have to do something together. Second, the related elements act toward meeting the functional requirements. Even if elements are related, they are not coordinated if they do not act toward meeting the functional requirements. Also, even if each element acts toward meeting the functional requirements, they are not coordinated if they are not related.

### Interrelation of Multiple Elements

Human body segments are mechanically linked by joints; therefore, the motion of one segment can be affected by the motion of other segments. Thus, it has been suggested that biomechanical analysis should consider the interrelation between segments rather than looking only at the kinematics of a single joint (Glazier and Robins, 2012; Glazier, 2017). Several studies have examined coupling, which provides a measure of the relative timing and magnitude of the motion between segments or joints (Heiderscheit et al., 2002; Ferber et al., 2005; Wilson et al., 2008). Coupling is used when, for example, the hip joint flexes or extends simultaneously with knee flexion or extension, referred to as hip-knee joint coupling. It is quantitatively evaluated by vector coding and relative phase analysis (Wheat and Glazier, 2006). Some studies refer to the interrelation of multiple elements as coordination, which is synonymous with coupling (Tepavac and Field-Fote, 2001; Abbasi et al., 2020; Acasio et al., 2021). However, coupling is different from biomechanical coordination. The fundamental difference is whether it includes meeting functional requirements. Coupling does not explicitly include meeting functional requirements and, therefore, may be irrelevant with regards to the achievement of a motor task.

Some studies used the term coordination to refer to muscle activation pattern (Prilutsky and Gregor, 2000; Larivière and Arsenault, 2008; Donath et al., 2015). The muscle activation pattern is defined as the repeated or regular way in which the muscle activates during a movement. Donath et al. (2015) examined the effects of aging and intense exercise on muscle activation patterns. Importantly, as with coupling, the muscle activation pattern does not explicitly include meeting functional requirements. In other words, patterned muscle activity does not necessarily act toward meeting functional requirements. However, confusingly, there are cases where the patterned muscle activity acts toward meeting the functional requirements. For example, during pedaling, the plantar flexors (gastrocnemius and soleus) and dorsiflexor (tibialis anterior) muscles show simultaneous activation, resulting in ankle stabilization (Raasch and Zajac, 1999; So et al., 2005). This stabilization increases the efficiency of energy transfer to the pedal and leads to pedal acceleration, which is a functional requirement of pedaling. Therefore, the relation between the plantar flexors and the dorsiflexor is interpreted as coordinating to meet the functional requirement of accelerating the pedal. Here, it is important to distinguish between the fact that the muscles show patterned activation and the fact that these muscles act toward meeting the functional requirements. This distinction avoids confusion between muscle activation patterns and biomechanical coordination.

### Bottom Line

When one term has multiple meanings, it can become confusing as in the case of coordination. We aimed to eliminate this confusion by clarifying the meaning of coordination in biomechanics. This required a conceptual analysis, which revealed that the coordination had at least two different meanings. The first refers to multiple elements related to achieving a common purpose, often referred to as synergy. The second refers to the interrelated elements. The former and the latter are distinctly different; the former requires that the interrelated elements act to meet functional requirements, while the latter does not. We believe that the careful application of this kind of analysis is relevant for the eradication of misunderstandings.

## Meaning of Coordination in Motor Control

### Computational Perspective

#### Coordination Related to Removing Redundancy

It is widely acknowledged that the human body has a greater number of degrees of freedom than necessary to successfully perform motor tasks (Bernstein, 1967). For example, for reaching a target in the two-dimensional space, the number of arm joint rotations is typically three (shoulder, elbow, and wrist joints). Thus, the position of the hand is determined by the combination of the three joint angles. However, the position of the hand is a single point in the two-dimensional space. Thus, there is not just one, but an infinite number of combinations of joint angles to determine the position of the hand. It is believed that the CNS is always faced with the problem of choosing a certain combination from an infinite number of possibilities (Franklin and Wolpert, 2011). This is known as the motor redundancy problem (Bernstein, 1967). When the brain is viewed as a computational machine, the need to instantly determine a certain combination from an infinite number of possibilities indicates that the CNS is required to perform an enormous amount of computation. The question of how the CNS deals with this problem has attracted the interest of researchers.

**Figure 2:**
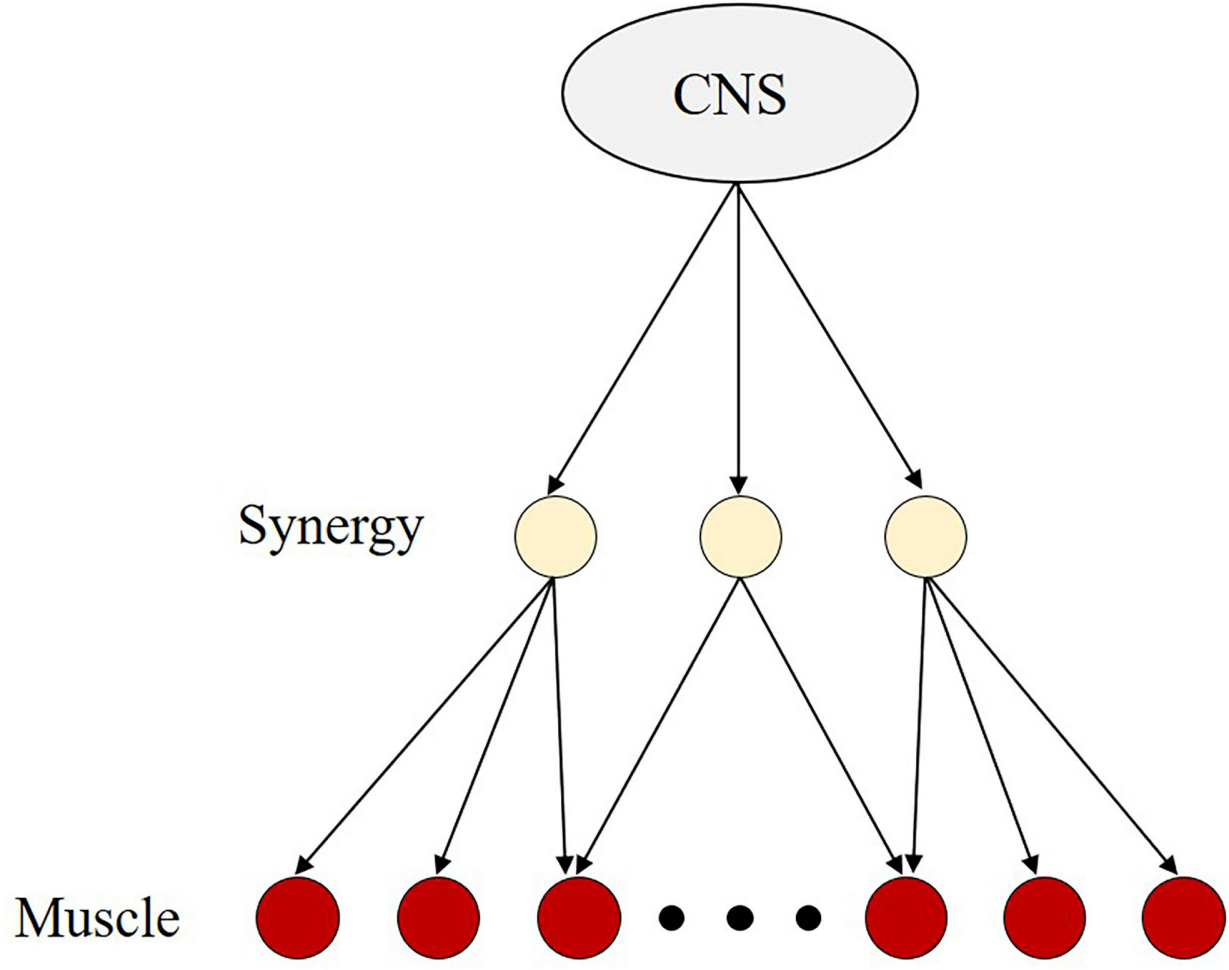
The concept of the muscle synergy hypothesis. Because the human body has a greater number of degrees of freedom to successfully perform the motor task, it is believed that the central nervus system (CNS) is always faced with the problem of choosing a certain combination from an infinite number of possibilities. It implies that the CNS needs to perform a large amount of computation. To address this problem, CNS is believed to adopt a control strategy that reduces the number of degrees of freedom requiring control, i.e., the muscle synergy hypothesis. The muscle synergy hypothesis is based on the assumption that the CNS controls synergies composed of multiple muscles, rather than individual muscles separately. With this control strategy, the CNS may be less computationally demanding than when controlling individual muscles.

The CNS is believed to address the problem by adopting a strategy to reduce the enormous amount of computation. The CNS does not control muscles individually but rather controls them *via* synergy which is a neural module that activates multiple muscles simultaneously (Saltiel et al., 2001; d'Avella et al., 2003; Chiovetto et al., 2013; Kuppuswamy and Harris, 2014) (Figure 2). One muscle can be part of multiple muscle synergies and one synergy can activate multiple muscles. This control strategy allows the CNS to reduce the number of degrees of freedom requiring control, thereby reducing the amount of computation. Then, the CNS can instantly determine a certain combination even in an infinite number of possibilities. In this context, the terms synergy and coordination have often been used synonymously (Bizzi et al., 2008; d'Avella and Lacquaniti, 2013).

The presence of synergy is shown using measured experimental data. A method for identifying synergy has been used to measure electromyographic signals of a large number of muscles and applies matrix factorization techniques (Tresch et al., 1999, 2006; d'Avella et al., 2003, 2006; Hart and Giszter, 2004; Ivanenko et al., 2004; Ting and Macpherson, 2005; Tresch and Jarc, 2009; Cheung et al., 2012). Ivanenko et al. (2004) demonstrated that the muscle activation of 12–16 ipsilateral leg and trunk muscles could be reduced to five basic independent components during walking. Additionally, d'Avella et al. (2006) identified four or five synergies in up to 19 arm muscles' activities in pointing movements. These studies concluded that muscle activation patterns were captured by a small number of time-varying synergies, suggesting that the CNS exploited this low dimensionality to simplify control.

Some researchers argue that muscle synergy reflects the basic aspects of muscle activation, but this argument has been criticized because there is a lack of direct evidence for the neural implementation of muscle synergy in the CNS (Hart and Giszter, 2010; Overduin et al., 2012; Cheung and Seki, 2021). This criticism suggests that muscle synergy is likely an artifact that relies on certain assumptions employed by the algorithm for separating the activations of the synergy. However, recent electrophysiological experiments have provided evidence that the activations of muscle synergy are expressions of neural activity (Hart and Giszter, 2010; Yakovenko et al., 2011; Overduin et al., 2012; Takei et al., 2017; Yaron et al., 2020). During voluntary hand movements, the muscle fields of premotor interneurons in the primate cervical spinal cord were not uniformly distributed across the hand muscles but rather distributed as clusters corresponding to muscle synergy. Using frog spinal cords, Hart and Giszter (2010) demonstrated that premotor interneurons have divergent output projections to motoneurons of muscles that match the muscle synergies identified from the electromyography of motor behaviors. These findings directly support the idea that muscle synergy is not an artifact but reflects the basic aspects of muscle activation.

#### Breaking Away From the View of Coordination as a Relation That Eliminates Redundancy

A different definition of synergy (often referred to as coordination) was provided at the end of the last century (Gelfand and Latash, 1998) and was later sophisticated by Latash and colleagues (Latash et al., 2007; Latash, 2012). They deviated from the view of synergy as a relation between elements to solve the computational problem on the CNS. Latash and colleagues described synergy as not related to a reduction of the amount of computation in the CNS. According to this view, it is assumed that the CNS does not produce a single optimal solution but provides families of combinations between elements that can achieve the motor task with acceptable accuracy. If the CNS determines a single solution from an infinite number of possibilities, an enormous amount of computation is required. Therefore, it is natural to assume that CNS will reduce the amount of computation. However, if the CNS provides a family of combinations that can achieve the motor task, the amount of computation required would be reduced. Therefore, there will be no need to eliminate the extra degrees of freedom. Latash and colleagues argue that having extra degrees of freedom than necessary means abundance, not redundancy (Latash et al., 2002; Gera et al., 2010; Latash, 2018). This abundance leads to multiple variations of the combinations between elements to achieve the motor task and is believed to allow for the flexibility to deal with unexpected perturbations applied to one or a few of the elements.

Latash and colleagues believe that the CNS does not strictly control individual joint movements, but focuses on control that minimizes variability in the outcome of motor tasks (in the case of a reaching task, the position of the hand). The CNS does not specify a precise pattern of joint movement that would lead to the desired outcome of motor tasks, but rather organizes the movement such that, if a certain joint movement introduces an error into the desired outcome, other joint movements compensate to minimize the original error. They defined the term synergy as a neural organization that ensures task-specific covariation of elements to contribute to the achievement of a motor task.

**Figure 3:**
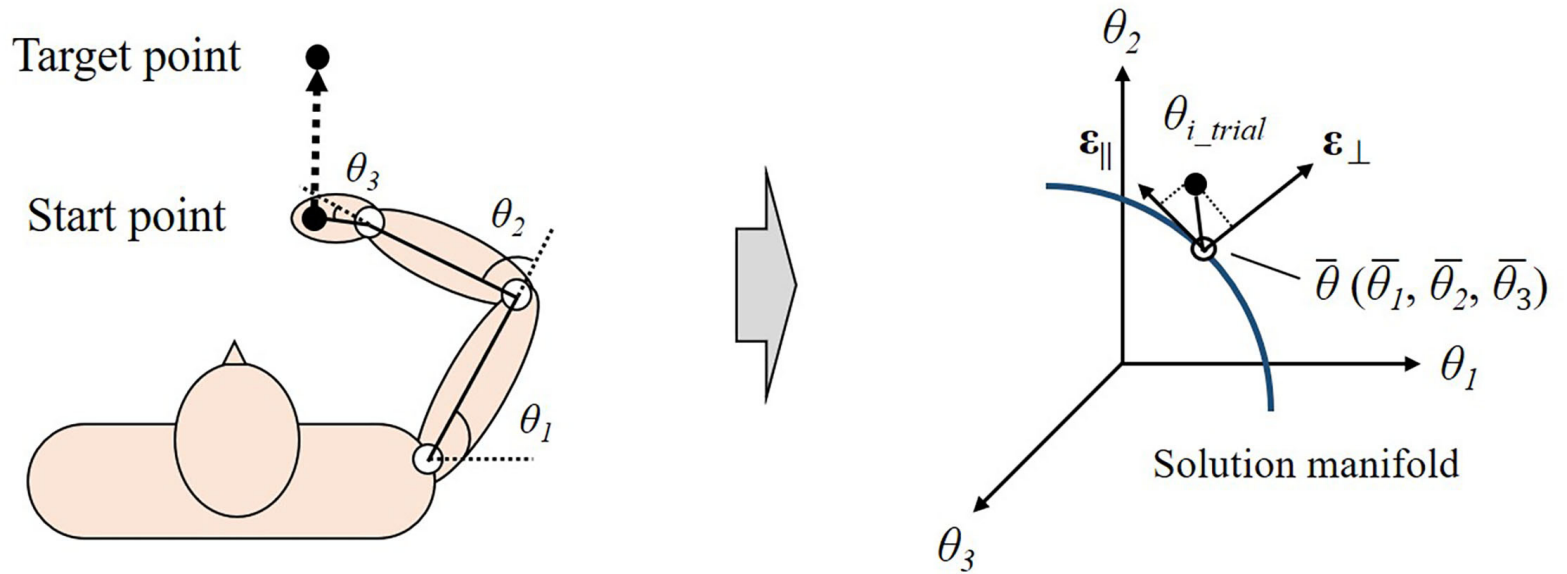
Overview of uncontrolled manifold (UCM) analysis in multidimensional space during reaching task. The state space is spanned by three variables (joint angles: θ_1_, θ_2_, θ_3_). The blue nonlinear line shows the solution manifold, the set of variables that achieve the task result with zero error. In a repetition of reaching task, the UCM analysis decomposed the variables of each trial (θ_*i_trial*_) into two components around the average (θ). One component lies parallel to the solution manifold (**ε_||_**) and the other component lies perpendicular to the solution space (ε_⊥_).

Synergy has often been examined by uncontrolled manifold (UCM) analysis (Scholz and Schöner, 1999; Scholz et al., 2000; Latash et al., 2001; Domkin et al., 2002, 2005; Krishnamoorthy et al., 2003; Tseng et al., 2003; Yang and Scholz, 2005) (Figure 3). This analysis determined the solution space of the motor task, which is a subspace related to achieving the motor task in the state space. The solution space is referred to as the uncontrolled manifold. The variables in the state-space can be divided into two orthogonal components. One component lies parallel to the solution manifold (||UCM), indicating that it does not deviate from the solution space. The other component lies perpendicular to the solution manifold (⊥UCM) and represents the configuration of variables away from the solution space. If the variability of ||UCM is higher than that of ⊥UCM when the same motor task is repeated, the variables are co-varied to achieve the motor task. In the study of Scholz et al. (2000), for instance, participants performed shooting with a laser pistol at a target. As a result, the seven joint angles of the arms co-varied to keep the orientation of the gun relative to the target invariant.

The UCM analysis is supposed to reflect a control hypothesis, i.e., the CNS controls variables that affect the achievement of a task (⊥UCM) and relatively does not control variables that do not affect the achievement of a task (|| UCM). In other words, the CNS selectively controls the variables that are not along the direction to the UCM. The presence of this control strategy has also been supported theoretically (Todorov and Jordan, 2002; Todorov, 2004; Diedrichsen, 2007; Liu and Todorov, 2007); the optimal feedback control model proposed by Todorov and Jordan (2002) does not act to correct movements in directions that have no bearing on the achievement of the motor task. The result is thought to suggest that the emergence of coordination reflects the operation of the control laws.

### Ecological Perspective

#### Self-Organized Coordination

Like the computational perspective, one of the most prominent issues in the ecological perspective is how the human body organizes the extra degree of freedom (Kay, 1988; Turvey, 1990; Van Emmerik et al., 2004). Coordination between elements may address this issue. This was first realized by Bernstein (1967), who believed that overcoming redundant degrees of freedom was the core of motor control. According to Bernstein, coordination was the reduction of a large number of degrees of freedom involved in a particular movement to a small number of variables. In the computational perspective, coordination is sometimes defined as the relation between elements that reduce the number of degrees of freedom controlled by the CNS. However, in the ecological perspective, the term coordination is defined differently than in the computational perspective. The coordination defined in the ecological perspective removes the need to consider the CNS even though it is associated with a reduction in the number of degrees of freedom (Vereijken et al., 1992; Turvey, 2007). The coordination between elements is not pre-programmed in the CNS but is a temporary functional grouping of elements that emerges without input from a controller *via* self-organizing processes. In this study, such coordination is called self-organized coordination. Self-organization is a process in which the order emerges solely from the interactions between elements and their surrounding environment, situation, and context (Haken, 2006). These interactions are performed without any external control.

**Figure 4:**
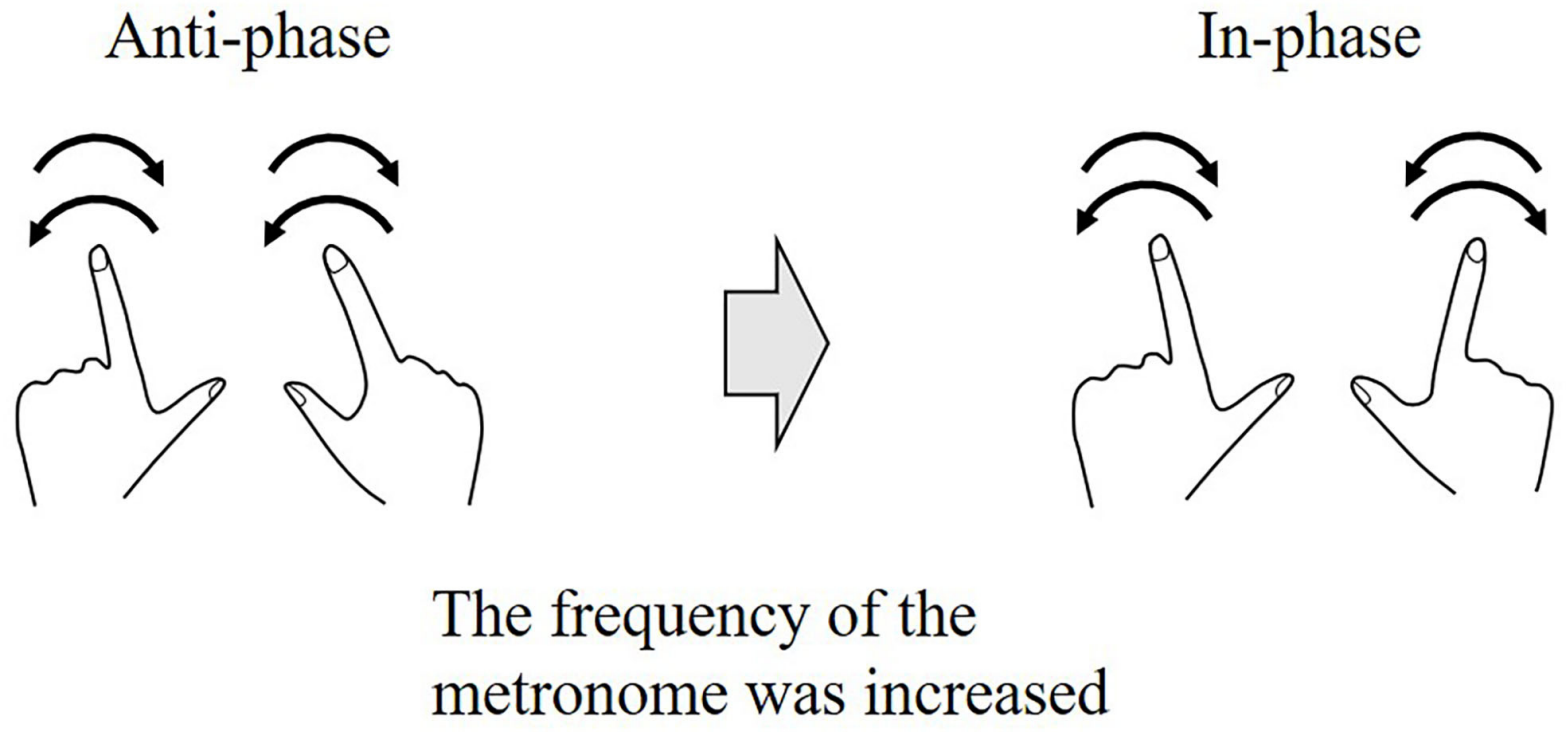
Phase transition during a rhythmical finger movement task. The experiment required participants to oscillate their index fingers to the frequency of the metronome in either in-phase or anti-phase. The participants oscillate their index fingers in the anti-phase pattern at a low frequency, and as the frequency was gradually increased, the phase pattern switches to an in-phase pattern at a certain critical frequency. This switching of the phase pattern is called a phase transition.

Self-organized coordination has been investigated using periodic movements. The experimental window into self-organized coordination was a paradigm introduced by Kelso et al. (1981), Kelso (1984). Kelso's original experiments dealt with rhythmical finger movement in a transverse plane (i.e., abduction/adduction) and required participants to oscillate their index fingers to the frequency of a metronome in one of two patterns, in-phase or anti-phase. In the in-phase pattern, both fingers move symmetrically (i.e., homologous muscle groups contracting simultaneously). In the anti-phase pattern, both fingers move asymmetry (i.e., homologous muscle groups contracting in an alternating fashion). These experiments showed that when participants began to oscillate their index fingers in the anti-phase pattern and the frequency of the metronome was gradually increased, the phase pattern became unstable and, at a critical frequency, suddenly changed to an in-phase pattern (Figure 4). These results are also supported by a theoretical model, the Haken-Kelso-Bunz (HKB) model (Haken et al., 1985). The experimental and model-based analyses showed the following two main findings. First, a single parameter, frequency, could manipulate the phase transition generated by the two-finger movement. Second, the instability or variability of the phase pattern promoted the spontaneous phase transition. This implies that the variability may be informative, not meaningless.

#### Coordination as a Correction of the Deviations in Elements

Insights gained from the ecological perspective have been successfully applied to a variety of fields. In particular, they have influenced the way that scientists view movement variability during repetition of a motor task (Hamill et al., 1999; Davids et al., 2003; Bartlett et al., 2007; Stergiou and Decker, 2011; Preatoni et al., 2013). According to traditional theory, movement variability was thought to be due to noise in the CNS. Thus, it was suggested that movement variability may emerge as an unwanted source of error that should be eliminated or reduced (Fitts, 1954; Schmidt et al., 1979; Harris and Wolpert, 1998; Van Beers et al., 2002). However, today, it is thought that movement variability does not only include an unwanted source of error (Müller and Sternad, 2003, 2004, 2009; Cohen and Sternad, 2009; Sternad, 2018). For example, in the reaching movement, the joint angles are slightly different in every trial. Despite this, the hand reaches the desired position and the reaching task is successful in every trial. It can therefore be expected that the joint angles are related such that the deviations in joint angles are canceled out, leading to a relatively invariant final hand position (Müller and Sternad, 2003). This relation between joint angles is sometimes termed coordination. Coordination means a relation that is beneficial to the achievement of a motor task and it differs from self-organized coordination.

**Figure 5:**
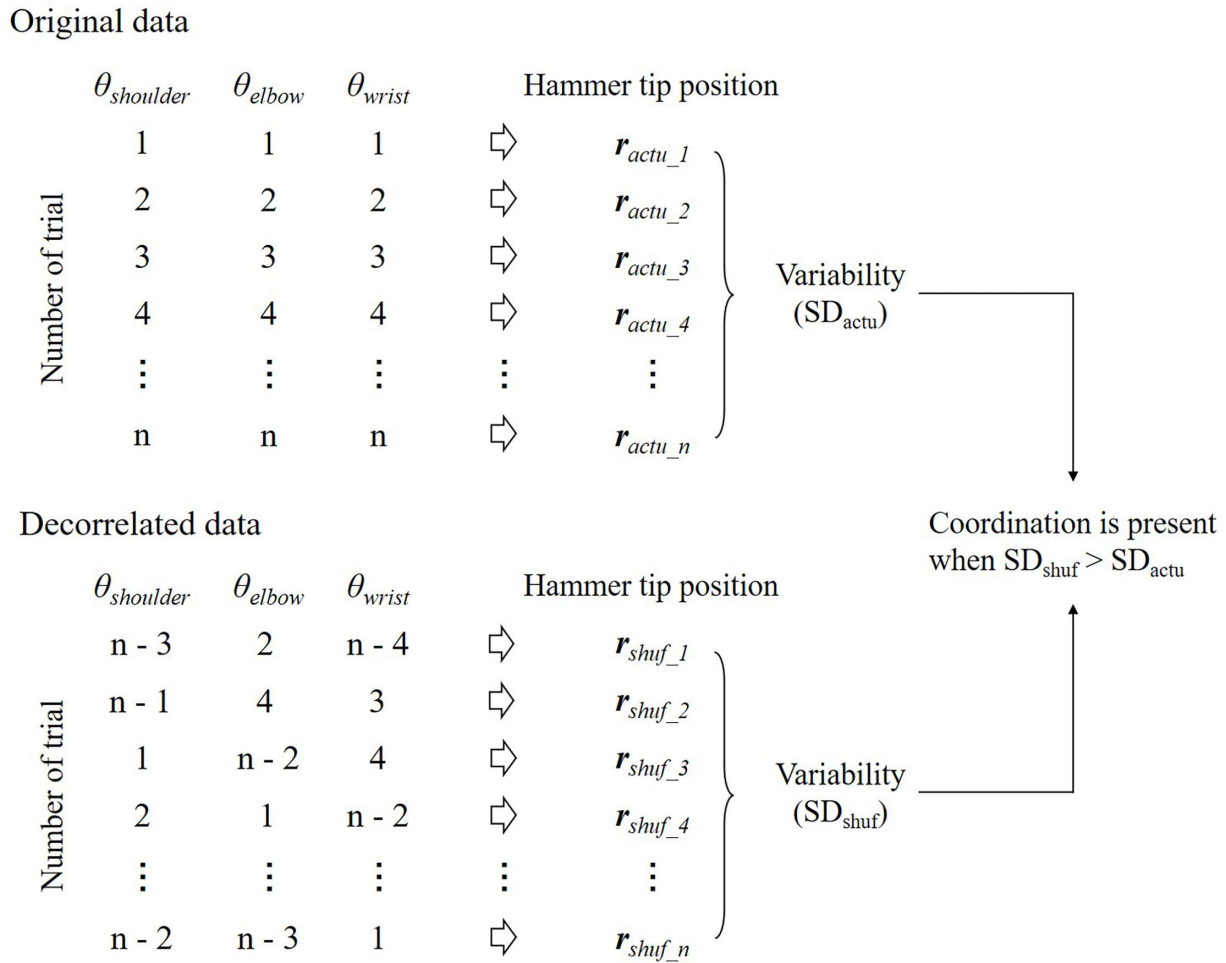
Illustration of the covariation by randomization (CR) analysis. All variables are randomly shuffled across trials to produce decorrelated data. This procedure allows for removing any correlation between variables within a trial. The decorrelated data are substituted into a forward dynamics model to determine the performance outcome and the standard deviation (SD) after the random shuffle (*SD*_*shuf*_). If *SD*_*shuf*_ is higher than the variability of the performance outcome for the original data (*SD*_*actu*_), the coordination between variables is present to reduce the variability of the performance outcome.

The presence of the coordination was first suggested by Bernstein's observation of hammering on an anvil (Bernstein, 1967). The participants were fully trained and had repeated the same movements hundreds of times a day for years. Nevertheless, he noticed that variability in the trajectory of the hammer tip in a series of strikes was smaller than the variability of the individual joint movements of the subject's arm holding the hammer; a famous phrase to describe this is “repetition without repetition.” Therefore, he suggested that the joints were not acting independently, but functionally linked to correct each other's errors. However, this was only suggestive and not conclusive. The variability in the spatial position of the tip of the hammer cannot be compared to the degree of reproducibility of the joint angle because these variables have different units. One way to solve this problem is covariation by randomization (CR) analysis (Kudo et al., 2000; Müller and Sternad, 2003, 2004; Verrel et al., 2010, 2012a,b) (Figure 5). CR analysis assesses coordination among joint angles by comparing the outcome of performance (e.g., the trajectory of the tip of the hammer) between the original and decorrelated data in a repetitive motor task. Decorrelated data are produced by randomly reordering joint angles across trials, thereby removing all possible correlations among them. Then, the decorrelated data are substituted into a forward dynamics model to determine the variability of the performance outcome. Thus, coordination is present when the variability of the performance outcome is higher for the decorrelated data than for the original data.

## Comparison Across Fields

### Biomechanics—Computational Perspective

Biomechanical coordination is the relation between elements that act toward meeting functional requirements and is examined to identify the function of elements involved in a particular movement (Table 1). On the other hand, coordination in the computational perspective sometimes means the relation between elements that reduce the number of degrees of freedom to solve a computational problem on the CNS and is examined to understand the control mechanism of the CNS (Table 1). Coordination was found to have different meanings. The finding leads to insights that biomechanical coordination is not necessarily related to reducing the CNS computation. For example, left and right hip abductor/adductor moments are coordinated to maintain a quiet standing, but this is not necessarily related to the reduction in the CNS computation. There may be cases where biomechanical coordination results in the reduction of the CNS computation, but that is not always the case.

**Table 1:** Meaning of coordination in each field.

**Field**	**Meaning of coordination**
Biomechanics	Relation that act toward meeting functional requirements Winter, 2009
Computational perspective	Relation that reduce the number of degrees of freedom to solve a computational problem on the CNS d'Avella et al., 2003
	Neural organization that ensures task-specific covariation of elements that contribute to the achievement of the motor task Latash et al., 2007
Ecological perspective	Self-organizing relation between elements that reduce degrees of freedom Turvey, 2007
	Relation that is beneficial to the achievement of a motor task Müller and Sternad, 2003

*The meaning of coordination and synergy within a field is similar although coordination is sometimes referred to as synergy*.

The meaning of coordination and the aim of studying coordination differ between biomechanics and motor control from the computational perspective, but it does not mean that these two fields are unrelated. Understanding the control mechanisms of the CNS, the aim of the computational perspective study, requires taking into account the biomechanics of the musculoskeletal system (Tytell et al., 2011). This is because there is no one-to-one correspondence between a neural command and the resulting movement (Zatsiorsky and Prilutsky, 2012). Even if the same neural command is input, the output movement differs depending on the current state of the musculoskeletal system (e.g., current joint angles and corresponding muscle moment arms, muscle lengths, and velocities). In addition, the state of the musculoskeletal system is physically determined by the behavior of neural circuits. These facts show that human movement is generated by the interaction between the behavior of neural circuits and the biomechanics of the musculoskeletal system. Thus, biomechanics and the computational perspective are closely related.

Latash and colleagues do not agree with the view that coordination is the relation between elements to reduce the number of degrees of freedom. Instead, they view coordination as a neural organization that ensures task-specific covariation of elements that contribute to the achievement of the motor task (Table 1). Such a covariation that contributes to the achievement of the motor task appears to be similar to biomechanical coordination. It may be the reason why UCM analysis is used not only in motor control but also in biomechanical studies (Verhoeven and Newell, 2016; Iino et al., 2017; Tokuda et al., 2018; Möhler et al., 2019, 2020). Verhoeven and Newell (2016) investigated the coordination between joint movements for the success of a basketball free-throw task using UCM analysis. They revealed that successfully controlling the trial-to-trial variability of release parameters (i.e., release position, angle, and speed) along the solution manifold and the coordination of postural control between joint movements changed as a function of skill level. It should be noted that the coordination used in these kinds of studies eliminates the consideration of the control by the CNS, while coordination as presented by Latash and colleagues considers the control of CNS.

### Biomechanics—Ecological Perspective

The prominent issue in the ecological perspective is how humans organize the extra degrees of freedom. The key concept for this is self-organized coordination, which is a temporary functional grouping of elements that emerges without input from a controller, such as the CNS (Table 1). Each element becomes functionally linked to behave as a task-specific unit, which reduces the number of degrees of freedom. It was found that biomechanical coordination and self-organized coordination have different meanings. This implies that even though self-organized coordination occurs between elements, it does not indicate that these elements act toward achieving a motor task. This can be explained using the rhythmical finger movement in a transverse plane example. This task required participants to oscillate their index fingers in anti-phase at the same frequency as a metronome. However, as the frequency of the metronome gradually increased, phase transition occurred at a certain critical frequency. This phase transition means that the purpose of the task is not achieved. Thus, the index fingers do not behave to achieve a motor task, although it is considered self-organized coordination.

Some researchers use the term coordination to mean covariation between elements that are beneficial to the achievement of a motor task (Müller and Sternad, 2003, 2004) (Table 1). These researchers are influenced by the notion that movement variability includes not only a meaningless but also a functional component. However, in the past, biomechanics researchers did not tend to consider movement variability as an important topic worthy of research attention (Glazier et al., 2006). This idea was derived from an implicit assumption commonly held by biomechanics researchers, namely that human movements were highly consistent when the same motor task was repeated (Glazier et al., 2006; Bartlett et al., 2007). Therefore, trial-to-trial variability was typically deemed to have negligible practical significance. However, even elite athletes show a certain amount of variability in movements (Davids et al., 2003), thus, this assumption is not valid. The trial-to-trial movement variability is not negligible, but rather noteworthy. Currently, the ecological perspective has been incorporated into biomechanics and it is accepted that variability has a functional component.

Since the idea of ecological perspective has been incorporated into biomechanics, the methods used in ecological perspective could be applied to biomechanics. However, simply applying the methods would be insufficient. Biomechanics researchers are interested in determining certain elements related to the achievement of a motor task (Lees, 1999); for this, the relation between the outcome of a performance (e.g., ball location, speed, etc. in throwing) and the individual elements (e.g. joint angle, angular velocity, etc.) are often examined (Chow and Mindock, 1999; Hughes and Bartlett, 2002; Chow and Knudson, 2011). Therefore, in biomechanics, it is valuable to examine which joint movements are coordinated for the achievement of a motor task. This cannot be addressed by simply using the methods employed in the ecological perspective, such as CR analysis. This is because CR analysis only shows the presence or absence of coordination to achieve the motor task and thus, does not provide information regarding which elements are coordinated. Applying methods from other related fields to biomechanics may require improvements of the methods to increase suitability for biomechanics (Kimura et al., 2021).

## Some Topics to be Discussed

Coordination is a growing interest in the field of biomechanics. This may be due to the idea that coordination is a fundamental concept for understanding what we perceive as the most basic of movements, such as locomotion, as well as complex movements in sports situations. In this study, we reviewed the main aspects of coordination in various fields and tried to clarify what coordination is in biomechanics. In this section, we present some topics to further promote coordination studies in biomechanics.

### Analytical Methods and Results Interpretation

Many methods have emerged to quantitatively evaluate coordination. UCM, principal component analysis (PCA) (Borghese et al., 1996; Bianchi et al., 1998; Ivanenko et al., 2007, 2008), CR, and relative phase (RP) analysis (Jeka et al., 1993; Kelso et al., 2001; Mechsner et al., 2001) are commonly used in motor control studies. Some of these methods are used in biomechanics. Worth mentioning is that the interpretation of the results differs depending on the study, even though the same method is used. Some studies interpreted the results obtained from UCM analysis as the CNS controlling variables that affect task achievement (⊥UCM) and relatively not controlling variables that do not affect task achievement (|| UCM) (Scholz and Schöner, 1999; Scholz et al., 2000; Latash et al., 2002; Tseng et al., 2003; Domkin et al., 2005; Schöner and Scholz, 2007). Scholz and Schöner (1999) concluded that, for the task of sit-to-stand, CNS predominantly controlled the position of the center of mass rather than the hand or head. Whereas, other studies interpreted that the results obtained from the UCM analysis indicated that the variables were related to achieving the motor task (Iino et al., 2017; Möhler et al., 2019; DiCesare et al., 2020). Iino et al. (2017) concluded that, for table tennis, the joint angles are related such that deviations in joint angles are canceled out to stabilize the racket angles at ball impact. The difference between these interpretations is whether the studies intended to understand the control of the CNS. The latter interpretation did not aim to understand the control of the CNS and therefore did not discuss the CNS even though they used UCM analysis. Failure to understand the differences in interpretation of results may lead to misunderstanding within the literature. Therefore, it is necessary to clarify not only the meaning of the concepts but also how to interpret the results.

### Links Between Biomechanics and Other Fields

Empirical examinations in biomechanics have focused on the analysis of elements that are related to the performance outcome (Chow and Mindock, 1999; Hughes and Bartlett, 2002; Chow and Knudson, 2011). These examinations enable coaches to know which elements to focus on (Chow and Knudson, 2011). As Glazier indicated, it is not enough for the biomechanical examination to analyze the correlations between performance outcomes and individual elements, but it is also necessary to analyze how the performance outcomes are produced by considering the interrelation between variables (Glazier and Robins, 2012; Glazier, 2017). However, biomechanical research has rarely considered interrelations and tends to describe what happens without explaining how performance outcomes are produced (McGarry, 2009; Glazier and Robins, 2012). These indications imply the importance of considering coordination in biomechanics. Indeed, while coaching focuses on a single part of the body, coaches know that changing the movement of one part affects the movement of other parts of the body. Therefore, to gain insights with practical implications, it is necessary to understand the fundamental relationship between a single focal movement and its relation to other movements.

The approach of focusing on interrelation is the main feature of network science (Newman, 2010). Network science is a field that studies networks of physical and biological phenomena, using methods including graph theory from mathematics and statistical mechanics from physics. In recent years, the theories and methods of network science have been applied not only to physics and biology but also to clinical biomechanics (Murphy et al., 2018). Murphy et al. (2018) viewed the musculoskeletal system as a network structure, composed of bones and the muscles that link them, and examined the clinical connections between structure and muscle injury. When a certain muscle is injured, other muscles may also be injured *via* compensatory mechanisms of the body (Colné and Thoumie, 2006). The effect of a certain muscle injury on the whole musculoskeletal system depends on how interconnections of the musculoskeletal system are structured. Therefore, it is believed that understanding these interconnections will help us to know which muscles are most at risk of secondary injury due to compensatory changes caused by focal injuries, thereby informing a more comprehensive approach to rehabilitation. In the future, it will be useful to explore the applicability of network science not only to clinical biomechanics but also to biomechanics with the aim of performance enhancement.

## Conclusion

Even though the term coordination has different meanings, few studies have summarized these meanings as we have. Therefore, it appears that the meaning of coordination has not been accurately understood. The present analysis of this study is not complete because it does not cover all of the literature, but we believe that it has organized the various theoretical standpoints on coordination and contributed to a more accurate understanding of coordination.

By mapping out several ideas regarding coordination, we hope that researchers will be able to make a conscious commitment to theoretical standpoints. Above all, we hope that this study will help researchers formulate their empirical arguments for coordination in a more transparent manner to improve the discussion. An inaccurate understanding of a concept may lead to errors in the interpretation of data and theory development. Finally, this study may help promote coordination studies in biomechanics.

## Author Contributions

AK was responsible for the conceptualization, investigation, writing—original draft, writing—review and editing, visualization, and funding acquisition. TY and HO were responsible for the visualization, writing—original draft, and writing—review and editing. All authors approved the final version of this manuscript.

## Funding

AK is supported by a grant from the Tateisi Science and Technology Foundation. The grant number is 2201007.

## Conflict of Interest

The authors declare that the research was conducted in the absence of any commercial or financial relationships that could be construed as a potential conflict of interest.

## Publisher's Note

All claims expressed in this article are solely those of the authors and do not necessarily represent those of their affiliated organizations, or those of the publisher, the editors and the reviewers. Any product that may be evaluated in this article, or claim that may be made by its manufacturer, is not guaranteed or endorsed by the publisher.
